# Interested, Though Not Competing

**Published:** 1912-03-16

**Authors:** A. Saxon Snell

**Affiliations:** 22 Southampton Buildings, Chancery Lane, London, W.C.


*To the Editor of* The Hospital.

### INTERESTED, THOUGH NOT COMPETING.

*To the Editor of* The Hospital.

Sir,—You have been good enough to forward me a marked copy of last week's issue of The Hospital in which attention is particularly drawn to some criticisms upon the award of this competition and reflections upon the system of judging generally.

The jury system, which appears so desirable to you, has been under consideration for many years by architects who are most intimately concerned in the matter. It has also been tried on more than one notable occasion, but the results have scarcely been such as to recommend it.

The whole system of competition is open to grave objection, although it must be acknowledged that it has some redeeming features. I venture to think, however, the pros and cons of a purely architectural question such as this is scarcely likely to be of interest to the majority of your readers. Neither is it necessary to trespass upon the space you may be good enough to allow me by dealing point by point with your correspondents' criticisms, which are so easy to make. They will be convincing to no one but such of the unsuccessful competitors who lack the instinct of true sportsmen.

I except one point, which, if left uncontradicted, may receive a certain amount of general credence. It is such a common grievance in competitions. Your correspondents . . . respectively state that :—

"The Committee fixed the sum of £35,000 (roughly £350 per bed) as the total cost for an up-to-date hospital," and "the cost was stipulated as being an essential in the selection, £35,000 being the sum the Committee would have available."

The best answer to these misleading statements is to quote the actual condition, which was as follows, viz. :—

"The Committee think that £35,000 is the maximum that they will have at their disposal for the whole hospital, and while they require the hospital to be up-to-date in all particulars, preference will be given to a design within that sum if possible, but competitors must use their discretion in designing if they think that amount insufficient."

No honest reading of this clause suggests that the question of cost was essential.

For the rest, no good award is ever made in consideration of one or two points, but on a general survey of the design from every point of view. In other words, the best design is that which combines the greatest number of good points and the least number of errors; and as to these, an assessor who has given many hours to the study of the plans must be a better judge than your anonymous F.R.I.B.A.'s. By the way, it would be interesting to have their names, and especially to know if either of them competed.

The assessor's character and his work as a hospital architect are too well known to be questioned, and your correspondents' criticisms will probably cause him more amusement than annoyance. I should like to take the opportunity of correcting a wrong but somewhat general impression. The authors of the successful design are my brother, Mr. John Saxon Snell, and Mr. Stanley M. Spoor, sometime my pupil, *not* your humble servant. Of the merits of their design it does not become me to speak, but I think no unprejudiced critic will dispute its general excellence.—Yours faithfully,

A. Saxon Snell.

22 Southampton Buildings,

Chancery Lane, London, W.C.,

